# Multiple molecular diagnoses identified through genome sequencing in individuals with suspected rare disease

**DOI:** 10.1016/j.xhgg.2025.100430

**Published:** 2025-04-07

**Authors:** Alka Malhotra, Erin Thorpe, Alison J. Coffey, Revathi Rajkumar, Josephine Adjeman, Naomi Dianne Naa Adjeley Adjetey, Sharron Aglobitse, Felix Allotey, Todor Arsov, Joyce Ashong, Ebenezer Vincent Badoe, Donald Basel, Yvonne Brew, Chester Brown, Kerri Bosfield, Kari Casas, Mario Cornejo-Olivas, Laura Davis-Keppen, Abbey Freed, Kate Gibson, Parul Jayakar, Marilyn C. Jones, Martina Kawome, Aimé Lumaka, Ursula Maier, Prince Makay, Gioconda Manassero, Marilyn Marbell-Wilson, Charles Marcuccilli, Diane Masser-Frye, Julie McCarrier, Hannah-Sharon Mills, Jeny Balazar Montoya, Gerrye Mubungu, Mamy Ngole, Jorge Perez, Eniko Pivnick, Milagros M. Duenas-Roque, Hildegard Pena Salguero, Arturo Serize, Marwan Shinawi, Fabio Sirchia, Claudia Soler-Alfonso, Alan Taylor, Lauren Thompson, Gail Vance, Adeline Vanderver, Keith Vaux, Danita Velasco, Samuel Wiafe, Illumina Laboratory Services Interpretation and Reporting Team, Ryan J. Taft, Denise L. Perry, Akanchha Kesari

**Affiliations:** 1 Illumina Inc., San Diego, CA, USA; 2 Genetic Alliance, Damascus, MD, USA; 3 Korle-Bu Teaching Hospital, Accra, Ghana; 4 Komfo Anokye Teaching Hospital, Kumasi, Ghana; 5 Ho Teaching Hospital, Ho, Ghana; 6 Goce Delcev Universiity, Stip, North Macedonia; 7 Cape Coast Teaching Hospital, Cape Coast, Ghana; 8 Children’s Wisconsin, Medical College of Wisconsin, Milwaukee, WI, USA; 9 Greater Accra Regional Hospital, Accra, Ghana; 10 University of Tennessee Health Science Center, Le Bonheur Children’s Hospital, Memphis, TN, USA; 11 Sanford Health, Fargo, ND, USA; 12 Neurogenetics Working Group, Universidad Cientifica del Sur, Lima, Peru; 13 Neurogenetics Research Center, Instituto Nacional de Ciencias Neurológicas, Lima, Peru; 14 Sanford USD Medical Center, Sioux Falls, SD, USA; 15 Children’s Hospital Los Angeles, Los Angeles, CA, USA; 16 Genetic Health Service, Wellington, New Zealand; 17 Nicklaus Children’s Hospital, Miami, FL, USA; 18 Rady Children’s Hospital, San Diego, CA, USA; 19 San Diego-Imperial Counties Developmental Services, Inc., San Diego, CA, USA; 20 Helensvale Medical Centre, Harare, Zimbabwe; 21 Center for Human Genetics, Universite de Kinshasa, Kinshasa, Democratic Republic of the Congo; 22 Holy Family Hospital, Techiman, Ghana; 23 Instituto Nacional de Salud del Nino-San Borja, Lima, Peru; 24 Mission Clinic, Accra, Ghana; 25 Rush University Medical Group, Chicago, IL, USA; 26 South Miami Hospital, South Miami, FL, USA; 27 Servicio de Genética, Hospital Edgardo Rebagliati Martins – EsSalud, Lima, Peru; 28 Padrino Children’s Foundation, Todos Santos, Baja California Sur, Mexico; 29 Washington University, St. Louis, MO, USA; 30 St. Louis Children’s Hospital, St. Louis, MO, USA; 31 Department of Molecular Medicine, University of Pavia, Pavia, Italy; 32 Medical Genetics Unit, IRCCS San Matteo Foundation, Pavia, Italy; 33 Texas Children’s Hospital, Houston, TX, USA; 34 Al Jalila Genomics Center of Excellence, Al Jalila Children’s Specialty Hospital, Dubai, United Arab Emirates; 35 Indiana University School of Medicine, Indianapolis, IN, USA; 36 Division of Neurology, Department of Pediatrics, Children’s Hospital of Philadelphia, Philadelphia, PA, USA; 37 Department of Neurology, Perelman School of Medicine, University of Pennsylvania, Philadelphia, PA, USA; 38 Point Loma Pediatrics, San Diego, CA, USA; 39 Children’s Hospital and Medical Center, Omaha, NE, USA; 40 Rare Disease Ghana Initiative, Accra, Ghana

**Keywords:** multiple molecular diagnoses, WGS, rare disease, secondary finding, incidental finding

## Abstract

Genome sequencing is a powerful and comprehensive test that detects multiple variants of different types. The interrogation of variants across the genome enables the identification of multiple molecular diagnoses (MMDs) in a single individual. In this retrospective study, we describe individuals in whom MMDs were associated with the proband’s indication for testing (IFT), secondary findings, or incidental findings. An MMD is considered where at least one of the findings is associated with the primary IFT and all variants are classified as either likely pathogenic or pathogenic. Clinical genome sequencing was performed for all individuals as part of the iHope program at the Illumina Laboratory Services between September 2017 and December 2023. The iHope cohort included 1,846 affected individuals, with 872 (47.2%) found to have at least one likely pathogenic or pathogenic variant associated with the primary IFT. Of these, 81 (9.3%) individuals had multiple clinically significant molecular findings, including 76 individuals with reported variants associated with 2 different conditions, and 5 individuals with more than 2 molecular findings. A total of 32 individuals (3.7%) had at least 2 molecular diagnoses related to the primary IFT, while in 49 (5.6%) individuals, the variant(s) reported for the second condition constituted a secondary or incidental finding. Our study highlights that among individuals with a likely pathogenic or pathogenic finding identified through genome sequencing, 9% have MMDs, which may have been missed with different testing methods. Of note, approximately 60% of the 81 individuals with an MMD had a potentially actionable secondary or incidental finding.

## Teaser

Genome sequencing enables the detection of multiple variants of different types and identification of multiple molecular diagnoses. Nine percent of individuals with a likely pathogenic or pathogenic finding had MMDs, which may have been missed with different testing methods. Approximately 60% of individuals with an MMD had a potentially actionable secondary or incidental finding.

## Introduction

Rare diseases affect approximately 300 million individuals worldwide, with the majority likely to have a genetic etiology but are as yet undiagnosed.^1^ These disorders represent a significant burden of disease globally and can have a complex etiology, with multiple underlying factors explaining an affected individual’s phenotype. Genome sequencing has been repeatedly shown to be a powerful single test that can detect multiple variant types, including single-nucleotide variants (SNVs), small insertions and deletions (indels), copy number variants (CNVs), mitochondrial variants, short tandem repeats (STRs), and some structural variants, thus providing a comprehensive evaluation of the genome and earning its place as a first-tier diagnostic test for rare diseases.^2^ Genome sequencing in individuals with rare diseases has shown a diagnostic yield ranging from 19% to 56%, depending on the cohort selection.^2^^,^^3^^,^^4^^,^^5^ With the comprehensive evaluation of the genome through genome sequencing, multiple molecular findings may be detected in a single individual.

Studies evaluating the presence of multiple molecular diagnoses (MMDs) in a single individual showed a range of 0.7%–7.5%.^6^^,^^7^^,^^8^^,^^9^^,^^10^^,^^11^^,^^12^^,^^13^^,^^14^^,^^15^ A majority of these studies used exome sequencing and focused mainly on findings overlapping the indications for testing (IFTs). Although MMDs would be expected to be higher for genome sequencing tests, to date only a few case reports^16^^,^^17^ and one large cohort^18^ have been described. The present report describes a thorough evaluation of MMDs identified through clinical genome sequencing (cGS) and describes MMDs that include not only those related to the proband’s phenotype but also secondary findings (SFs) and incidental findings (IFs). The individuals in this cohort were suspected to have a rare genetic disease and belonged to 24 global clinical sites from the United States, Mexico, Peru, the Democratic Republic of Congo, Ghana, Italy, New Zealand, and the United Arab Emirates.^2^

## Subjects and methods

This retrospective study was conducted on individuals with suspected rare genetic diseases, who received cGS through the Illumina Laboratory Services between September 2017 and December 2023. Testing was provided to families at no cost through the iHope program, a philanthropic clinical implementation program to reduce access barriers to molecular genetic testing for underserved children globally with a suspected rare disease. For some of the participating sites, genome sequencing was the first line of testing in many of the participants, and no prior tests such as karyotype or microarray had been conducted. Retrospective analysis of test outcomes from the iHope program was reviewed by the WIRB-Copernicus Group, which granted an institutional review board exemption with a Health Insurance Portability and Accountability Act Full Waiver of Authorization as defined in the US Department of Health and Human Services’ 45CFR46.104(d) (4). cGS was pursued for all individuals as previously described.^2^ Briefly, the cGS test interrogated SNVs, small indel events, CNVs, homozygous loss of *SMN1,* mitochondrial SNVs, and a set of 21 STR expansions with known associations with genetic disorders. Reported variants in regions known to have homologous sequences were orthogonally confirmed before reporting.

For this study, MMDs are defined as the presence of at least two variants associated with different conditions, present in the molecular state expected to cause disease, with at least one condition overlapping the IFT. Variants must be classified as likely pathogenic or pathogenic (LP/P) using the American College of Medical Genetics and Genomics (ACMG) classification framework^19^ for the disorder to be included as an MMD, with an exception for autosomal recessive conditions. For autosomal recessive conditions, potential diagnoses with compound heterozygous variants associated with recessive conditions in which one variant is LP/P and the second variant is a variant of uncertain significance (VUS) were included. For all individuals who opted in for SF testing, SF genes were evaluated for clinically significant variants in the list of genes as recommended by ACMG, which included 55, 59, 73, or 78 genes, depending on the time of testing.^20^^,^^21^^,^^22^ In addition, IFs in genes that are not included in the SF list but meet defined actionability criteria either through screening or treatment options, or in rare instances if the associated condition manifests in early childhood, were reported and considered in this study.^23^ Of note, STRs that do not overlap with the proband’s phenotype were not reported as IFs.

## Results

**Figure 1:**
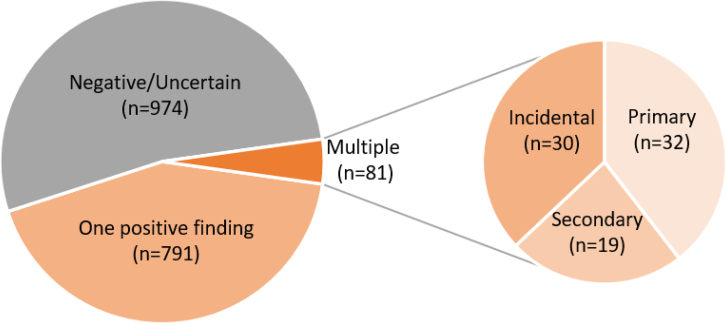
Breakdown of report outcome for all cases (*N* = 1,846) Negative report issued = 974; positive report issued with single finding = 791; positive with multiple findings = 81, including 32 with multiple primary findings, 19 with primary and secondary findings, and 30 with primary and incidental findings.

**Figure 2:**
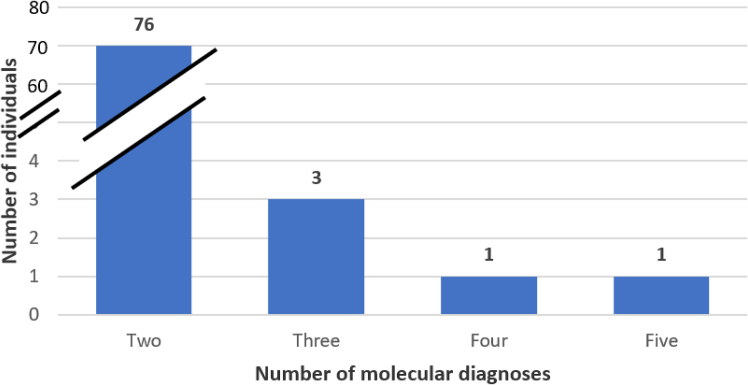
Number of molecular diagnoses identified per individual among the 81 individuals

Genome sequencing was performed in a total of 1,846 unrelated individuals suspected to have a rare genetic disease.^2^ A total of 872 individuals (47.2%) had a positive report, with at least one variant classified as likely pathogenic/pathogenic in a disease-associated gene(s) consistent with the clinical presentation (Figure 1). Of these, 81 (9.3%) had MMDs (Figure 2; Table S1).

The MMDs were categorized based on phenotype overlap and showed 32/81 individuals (39.5%) with multiple variants in genes associated with conditions overlapping the IFT (Figure 1). A complete list of conditions can be found in Table S1. Thirty of the 81 individuals (37%) had at least one primary and one IF (Figure 1). This included two individuals with the well-described pathogenic mitochondrial variant m.1555A>G in *MT-RNR1*, which is associated with hearing loss with or without exposure to aminoglycosides. Most of the identified variants were rare, but some more common variants were also reported given the actionability of the condition based on the framework for reporting IFs described by Brown et al.^23^ This includes variants in the *G6PD* gene, which is associated with glucose-6-phosphate dehydrogenase (G6PD) deficiency. Pathogenic variants in this gene were identified in 15 individuals (50% of IF), mainly of African ancestry in the iHope cohort, including 8 males in a hemizygous state, 6 females in a heterozygous state, and 1 female in a homozygous state. SFs were identified in 19/81 (23.5%) individuals in 16 different genes mainly associated with hypercholesterolemia, cardiac phenotypes, and cancer susceptibility. It should be noted that some cases that fell in the IF group would now be part of the SF list (e.g., disease-causing variants in *TTR* were reported as IFs in this cohort given that *TTR* was only recently added to the SF version 3.1 list).^21^

Five individuals (6.2% of those with MMDs) have more than two molecular diagnoses (Table 1). One individual (previously published)^24^ had five molecular diagnoses, with three overlapping the primary phenotypes, including SNVs in *SCN2A* and *KISS1R* and a large deletion encompassing *KISSR1* (associated with the proband’s phenotype) and *STK11* (SF). In addition, this individual had an IF in *CHEK2* and a mosaic uniparental disomy of the 11P region associated with Beckwith-Wiedemann syndrome. We note that one of the variants in *KISSR1* is classified as VUS, but this individual still has MMDs, given the other LP/P findings. Another individual had three CNVs, including deletions of chromosome 1p21.1 and Xq22.1 and a duplication of Xp21.1, all associated with the primary phenotype (Table 1).

**Table 1:** Individuals with more than two variants classified as likely pathogenic or pathogenic associated with at least two distinct conditions

Individual	Clinical features	Variant details	Associated condition	Primary/secondary/incidental findings	Variant classification
1	severe growth deficiency, epilepsy, developmental delay, lack of speech, behavioral issues, not-developed secondary sexual characteristics, coarse facies, long tapered fingers, thick lips, over-folded ears, broad-based duck-footed gait, lactose intolerance, constipation	*SCN2A* (NM_001040142.1): c.224C>G; p.Ser75Ter	*SCN2A*-related seizures	primary	P
		*CHEK2* (NM_007194.3): c.349A>G; p.Arg117Gly	Cancer susceptibility	incidental	LP
		*KISS1R* (NM_032551.4): c.233A>G; p.Asn78Ser	Isolated gonadotropin-releasing hormone deficiency	primary	VUS^a^
		CNV with *KISSR1* and *STK11* full gene deletion	Isolated gonadotropin-releasing hormone deficiency; Peutz-Jeghers syndrome	primary; secondary	P
		11p15 mosaic uniparental disomy	Beckwith-Wiedermann syndrome	incidental	P
2	pulmonary hypertension, cleft palate, mild micrognathia, prominent forehead, high triglycerides, history of 2-vessel cord prenatally	1p21.1 deletion	1p21.1 deletion	primary	LP
		Xq22.1 deletion	Turner syndrome	primary	P
		Xp21.1 duplication	Dystrophinopathy	primary	P
3	microcephaly, truncal hypotonia, syndactyly of the hands and right foot, dysgenesis of the corpus callosum, feeding difficulties, failure to thrive, patent ductus arteriosus, developmental delay, low anterior and posterior hair lines, long eyelashes, bushy arched eyebrows, depressed and broad nasal bridge, small jaw, thin upper lip	*NAA15* (NM_057175.3): c.1477C>T; p.Gln493Ter	Intellectual developmental disorder	primary	P
		*EP300* (NM_001429.3): c.5688_5689delGT; p.Ser1897ProfsTer3	Rubinstein-Taybi syndrome	primary	P
		15q13.2-q13.3 duplication	15q13.3 BP4-BP5 duplication	primary	P
4	pre- and post-natal growth failure, microcephaly, short stature, developmental delay, impaired cognitive function, sensory processing difficulty, high arched palate, ankyloglossia, facial muscle weakness, asymmetrical face, micrognathia, congenital malrotation of intestine, hypospadias, sacral pit, clinodactyly, cutis marmorata	*RAD21* (NM_006265.2): c.233_234delGT; p.Cys78Ter	Cornelia de Lange syndrome	primary	LP
		*FGG* (NM_021870.2): c.323C>G; p.Ala108Gly	Congenital fibrinogen deficiency	primary	LP
		*SDHA* (NM_004168.3): c.1534C>T; p.Arg512Ter	Hereditary paraganglioma-pheochromocytoma syndrome	incidental	P
		*ZC4H2* (NM_018684.3): c.431C>G; p.Thr144Arg	*ZC4H2*-related X-linked intellectual disability	primary	VUS^a^
5	downturned corners of mouth, anemia, right atrial enlargement, narrow chest, secundum atrial septal defect, microphthalmia, neurodevelopmental delay, short neck, numerous nevi, abnormal hemoglobin, overlapping toe, single transverse palmar crease, preauricular skin tag, telecanthus, craniosynostosis, pulmonary artery hypoplasia, plagiocephaly, downslanted palpebral fissures, webbed neck, recurrent pneumonia, right ventricular hypertrophy, anal fistula, hypertelorism, broad neck, microcephaly, pectus excavatum, hydronephrosis	*HBB* (NM_000518.4): c.19G>A; p.Glu7Lys *HBB* (NM_000518.4): c.20A>T; p.Glu7Val	Sickle-hemoglobin C disease deficiency	primary	P; P
		*SGCE* (NM_003919.2): c.45G>A; p.Trp15Ter	DYT-SGCE	incidental	LP
		*G6PD* (NM_000402.4): c.[292G>A; 466A>G]; p.[Val98Met; Asn156Asp]	G6PD	incidental	P
		11q23.3-q25 deletion	Jacobsen syndrome	primary	P

Additional reported variants, including those classified as VUS, are also listed to provide the complete information included in the clinical report. G6PD, glucose-6-phosphate dehydrogenase; DYT-SGCE, SGCE-related dystonia; LP, likely pathogenic; MMD, multiple molecular diagnoses; P, pathogenic; VUS, variant of uncertain significance.

a Some VUS variants were also reported and are listed here to provide the complete information included in the clinical report; these individuals still meet the criteria for an MMD based on the other LP/P findings.

**Figure 3:**
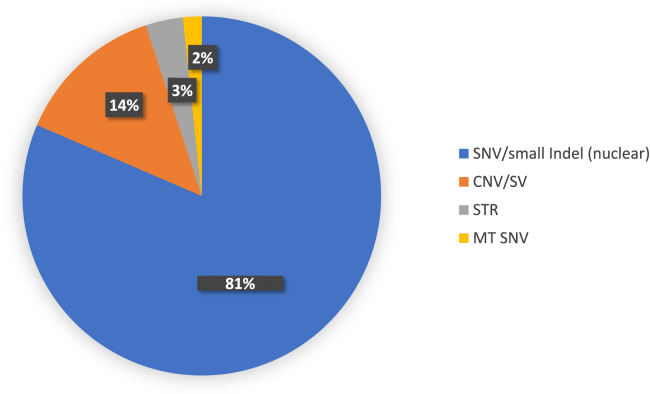
Variant types and overall percentage detected in individuals with multiple molecular findings CNV, copy-number variant; MT, mitochondrial variant; SNV, single-nucleotide variant; STR, short tandem repeat; SV, structural variant.

Evaluation of the variant types showed a majority were small variants, including 145 LP/P nuclear DNA variants and three mitochondrial variants (this includes the same variant identified in two individuals). Expanded STRs associated with different forms of spinocerebellar ataxia together with a small nuclear DNA variant were reported in six individuals. In addition, 24 CNVs or structural variants were identified in these individuals (Figure 3). Across the different variant types, 22.4% of the variants contributing to the MMDs were *de novo*.

## Discussion

MMDs are a frequent occurrence and likely to be underestimated in some studies. The use of genome sequencing makes it more likely to detect MMDs compared to other genetic tests currently available. Our study describes individuals in whom multiple LP/P variants detected in the molecular state were expected to cause disease, with at least one of the variants being associated with the IFT. Taking primary, SFs, and IFs into consideration, 9.3% of all cases with a positive finding had multiple diagnoses. Of these, 32 individuals had multiple variants associated with the IFTs only, and 49 individuals had SFs and/or IFs in addition to at least one primary finding. Of note, several of these MMDs were a result of identification of multiple variant types. For example, individual 3 (Table 1) had two small variants (stop gained and frameshift variants in *NAA15* and *EP300*, respectively) and one CNV (duplication of chromosome 15q13.2-q13.3), all overlapping the proband’s phenotype. The identification of multiple variant types in a single individual shows the benefit of cGS as a comprehensive test. Some of the variants, for example, large CNVs or structural variants, would likely be missed through single-gene and panel tests; multiple tests would be needed to be able to identify such diverse variant types. Furthermore, inclusion of STRs and mitochondrial variants would not be captured through exome sequencing. In the present study, six individuals were identified with STRs and three with mitochondrial variants who would not have been identified through exome sequencing alone. Exclusion of these individuals would result in a lower number of individuals with MMDs.

Previous studies evaluating exome sequencing in individuals from rare disease cohorts found a range of 0.7%–7.5% of individuals with MMDs, all of which mainly included variants in genes associated with the primary indication. Some of these studies also included VUSs.^6^^,^^7^^,^^8^^,^^9^^,^^10^^,^^11^^,^^12^^,^^13^ Two large exome cohort studies with >2,500 individuals showed an average rate of MMDs of 2% and 3.5%, respectively.^6^^,^^8^ Smith et al.^8^ noted that the rate of multiple molecular findings was slightly higher in individuals with complex phenotypes associated with multiple organ systems compared to those with involvement of single systems. Similarly, consanguineous families showed higher rates compared to nonconsanguineous families.^8^^,^^9^ Recently, Guo et al.^18^ used genome sequencing to evaluate MMDs by assessing multiple variant types. In the Guo et al.^18^ study, MMDs overlapping the proband’s phenotype were identified in 15 of 273 individuals (5.5%), with at least one molecular diagnosis where the variants were classified as LP/P, compared to 3.7% (32/872) in the present study. The cohort, population demographics, and type of conditions present in these individuals may explain the differences in the number of MMDs identified between these studies.

The iHope cohort includes study sites in geographic regions that were not evaluated by previous exome studies, for example, Ghana and the Democratic Republic of the Congo. In these populations, specific variants had a higher occurrence, including some common pathogenic *G6PD* variants that were reported as IFs, which contributed to the increase in MMDs.

Prior studies investigating multiple diagnoses to date focused on those related to the IFT only, with the exception of Guo et al.,^18^ in which nine individuals were identified with one variant related to the primary phenotype and one SF. In addition, as noted above, some studies also counted VUS variants, while in the present study, only LP/P variants are considered for autosomal dominant conditions, and at least one of the pair is required to be LP/P for an autosomal recessive condition. These differences across studies may also contribute to the variability in the overall frequency of MMDs.

With the inclusion of IFs and SFs, we identified additional variants potentially relevant to the clinical management of the patient. The potential for clinical utility of an actionable finding in the patient and family members was observed in 49 individuals, all with at least one primary finding and at least one SF or IF. For example, individuals can be monitored and regularly screened for cancer-related phenotypes associated with *CHEK2* and *STK11* (individual 1) and *SDHA* (individual 4). IFs were reported in 30 individuals and all were deemed actionable. Common *G6PD* variants were identified in 15 individuals in our study, mainly of African descent. Hemizygous males and homozygous females for a pathogenic variant are considered to be G6PD deficient, while heterozygous females may show a range of severity for G6PD deficiency.^25^ The return of this result is important, given that the avoidance of certain medications and foods, including fava beans, can prevent life-threatening outcomes. In addition, the level of deficiency needs to be considered, especially given the wide range of G6PD deficiency in heterozygous females.^25^^,^^26^

Because the study involved the evaluation of data from 2017 to 2023, some aspects of the analysis workflow varied over the years. For example, genes added to later versions of the ACMG SF gene list^21^^,^^22^ were not retrospectively evaluated in all individuals who opted in to the SF analysis. Similarly, reporting practices regarding IFs naturally evolved over time.^23^ Therefore, there is a potential for more MMDs to have been discovered in this cohort. The benefits of reanalysis of genome sequencing data are well established in terms of increasing diagnostic rates given improvements in sequencing technologies, bioinformatic tools, and additional information in biological and clinical databases, particularly new gene-disease relationships. Retrospective reanalysis for all cases is beyond the scope of this study. A systematic reanalysis could result in an increase in the number of MMDs.

This study describes an in-depth evaluation of MMDs identified through genome sequencing. Variants were reported in genes associated with the primary IFTs as well as IFs and SFs, with the latter two categories providing additional actionability that could allow a change in management for the proband and family members. This study highlights the need for comprehensive testing provided by genome sequencing to allow the capture of MMDs that will enhance patient care in individuals with rare diseases.

## Data and code availability

The data supporting the present study have not been deposited in a public repository to ensure anonymity and privacy. De-identified outcomes data detailed in this paper can be used for further study. Clinical sequencing data will not be made available to external researchers, given the privacy and protections inherent in a clinical sequencing test. Per routine laboratory practice, Illumina Laboratory Services reported variants identified in the iHope population to ClinVar: https://www.ncbi.nlm.nih.gov/clinvar/submitters/504895/.

## Consortia

Illumina Laboratory Services Interpretation and Reporting Team members: Subramanian S. Ajay, Laura M. Amendola, Max Arseneault, James Avecilla, Anusha Balusu, Maren Bennett, Krista Bluske, Carolyn Brown, Matthew P. Brown, Amanda Buchanan, Brendan Burns, Nicole J. Burns, Anjana Chandrasekhar, Aditi Chawla, Amanda Clause, Katie Golden-Grant, Vlad Gainullin, R. Tanner Hagelstrom, Rueben Hejja, Basil Juan, Josh Lowry, Philip Medrano, Becky Milewski, Felipe Mullen, Viswateja Nelakuditi, Julia Ortega, Vani Rajan, Ajay Ramakrishnan, Samin A. Sajan, Alicia Scocchia, Zinayida Schlachetzki, Sarah Schmidt, Samuel Strom, Julie P. Taylor, Brittany Thomas, Pratyusha Vankayala, and Sylwia Urbaniak.

## Acknowledgments

The authors are grateful to all the individuals and families who participated in this study. We also thank John Belmont, Keisha Robinson, Bright Dunyo, and the Illumina Laboratory Services customer support and bioinformatics teams.

## Author contributions

A.M., E.T., A.J.C., and R.R.: conceptualization, investigation, data curation, writing – original draft. A.K., D.L.P., and R.J.T.: conceptualization, supervision, writing – review & editing. Illumina Laboratory Services Interpretation and Reporting Team: investigation. J.A., N.D.N.A.A., S.A., F.A., T.A., J.A., E.V.B., D.B., Y.B., C.B., K.B., K.C., M.C.-O., L.D.-K., A.F., K.G., P.J., M.C.J., M.K., A.L., U.M., P.M., G.M., M.M.-W., C.M., D.M.-F., J.M., H.-S.M., J.B.M., G.M., M.N., J.P., E.P., M.M.D.R., H.P.S., A.S., M.S., F.S., C.S.-A., A.T., L.T., G.V., A.V., K.V., D.V., and S.W.: resources, writing – review & editing.

## Declaration of interests

A.J.C., A.K., A.M., D.L.P., E.T., R.J.T., R.R., and the Illumina Laboratory Services Interpretation and Reporting Team are either current or former employees and shareholders at Illumina, Inc.

## Supplemental information

Table S1. Clinical and molecular details of individuals with multiple molecular diagnoses
